# Multiple paternity in two populations of finetooth sharks (*Carcharhinus isodon*) with varying reproductive periodicity

**DOI:** 10.1002/ece3.7948

**Published:** 2021-07-29

**Authors:** Cody S. Nash, Philip C. Darby, Bryan S. Frazier, Jill M. Hendon, Jeremy M. Higgs, Eric R. Hoffmayer, Toby S. Daly‐Engel

**Affiliations:** ^1^ Department of Biology University of West Florida Pensacola FL USA; ^2^ South Carolina Department of Natural Resources Marine Resources Research Institute Charleston SC USA; ^3^ Center for Fisheries Research & Development Gulf Coast Research Laboratory The University of Southern Mississippi Ocean Springs MS USA; ^4^ National Oceanic and Atmospheric Administration National Marine Fisheries Service Southeast Fisheries Science Center Mississippi Laboratories Pascagoula MS USA; ^5^ Department of Ocean Engineering and Marine Sciences Florida Institute of Technology Melbourne FL USA

**Keywords:** elasmobranchs, mating strategies, polyandry, population genetics

## Abstract

The mechanisms underlying polyandry and female mate choice in certain taxonomic groups remain widely debated. In elasmobranchs, several species have shown varying rates of polyandry based on genetic studies of multiple paternity (MP). We investigated MP in the finetooth shark, *Carcharhinus isodon*, in order to directly test the encounter rate hypothesis (ERH), which predicts that MP is a result of the frequency of encounters between mature conspecifics during the breeding season, and should therefore increase when more time is available for copulation and sperm storage. Female finetooth sharks in the northern Gulf of Mexico (GoM) have been found to reproduce with both annual periodicity and biennial periodicity, while finetooth sharks from the northwestern Atlantic Ocean have only been found to reproduce biennially, allowing us to compare mating opportunity to frequency of MP. Our results show high rates of MP with no significant difference in frequency between females in the GoM (83.0%) and Atlantic (88.2%, *p* = .8718) and varying but nonsignificant rates of MP between females in the GoM reproducing annually (93.0%) and biennially (76.6%, *p* = .2760). While the ERH is not supported by this study, it remains possible that reproductive periodicity and other physiological factors play a role in determining rates of MP in elasmobranchs, with potential benefits to individuals and populations.

## Graphical abstract

We investigated multiple paternity in the finetooth shark in order to directly test the encounter rate hypothesis, which predicts that multiple paternity is a result of the frequency of encounters between mature conspecifics during the breeding season. We found high rates of multiple paternity with no significant differences corresponding to reproductive periodicity or location.

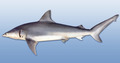

## 1 INTRODUCTION

Multiple mating (mating with more than one conspecific over the course of the reproductive season) by one or both sexes is common to many animal groups (Zeh & Zeh, 2003). For males, fitness hypothetically improves through remating with multiple females (polygamy) because of the increased likelihood of siring more offspring combined with the low cost of producing sperm compared with eggs (Bateman, 1948). Conversely, the benefits of females mating with multiple males (polyandry) are less obvious, but such behavior is more common in nature than previously thought, having been found in many taxa including all classes of vertebrates (reviewed in Taylor et al., 2014). It is hypothesized that polyandry may benefit populations by maximizing effective population size (*N*_e_) and maintaining high levels of genetic diversity (Pearse & Anderson, 2009). If true, then species with a higher frequency of polyandry should have a better chance of recovering from population decline, though it has also been suggested that polyandry may actually lower genetic diversity and *N*_e_ by increasing the variance in male reproductive success (Karl, 2008; Lotterhos, 2011).

The advancement of genetic techniques has made studying female mating strategies in nature more feasible because of the ability to detect polyandry via multiple paternity (when a single brood is sired by more than one male) using DNA analysis of the female and offspring (Jones et al., 2010). Molecular studies in the past 20 years have revealed a surprising amount of polyandry in systems that were expected to be genetically monogamous, especially where social monogamy was observed, such as in passerine birds (Petrie & Kempenaers, 1998) and mammals (Thonhauser et al., 2013), where females invest heavily in reproduction and are therefore expected to derive little benefit from remating (Zeh & Zeh, 2001). Recently, the genetic mating systems of elasmobranchs (sharks, skates, and rays) have garnered interest because of growing concern for shark population persistence and conservation (Dulvy et al., 2014). Elasmobranchs have a relatively wide variety of reproductive strategies for a large vertebrate, ranging from oviparity (egg‐laying) to several forms of viviparity (live birth), including placental viviparity (Parsons et al., 2007; Pratt & Carrier, 2001). All elasmobranchs use internal fertilization, and females invest heavily in reproduction compared with males through long gestation periods (4.5–36 months) and large, energetically expensive young (Conrath & Musick, 2012; Tanaka et al., 1990). Yet, polyandry leading to multiple paternity has been shown to be highly common in elasmobranchs and thought to be facilitated by female sperm storage (Fitzpatrick et al., 2012), which is common and can persist in some species for years (Conrath & Musick, 2012; Pratt & Carrier, 2001). Although frequency of multiple paternity has been shown to vary widely both within and between species in a way that implies local adaptation (Chabot & Haggin, 2014; Daly‐Engel et al., 2007, 2010; Fitzpatrick et al., 2012), the direct behavioral causes and potential ultimate evolutionary benefits of this behavior in sharks remain unclear.

Several hypotheses attempt to explain the adaptive significance of multiple paternity in sharks (reviewed in Fitzpatrick et al., 2012). Multiple mating has obvious benefits to male fitness since males will likely sire more offspring with each additional mate. Conversely, polyandrous mating behavior by female sharks may actually decrease their fitness as a result of wounds inflicted during copulation, when males are known to grasp the flanks and pectoral fins of the females with their teeth (Pratt & Carrier, 2001). As a result, the study of multiple mating in sharks has long focused on the role of females and female choice (Daly‐Engel et al., 2010; Fitzpatrick et al., 2012). Multiple paternity may be favored to evolve in species in which the female has a lower risk of injury if she submits to copulation, a hypothesis known as convenience polyandry (DiBattista et al., 2008). Alternatively, polyandry may be a function of female mate choice, either pre‐ or postcopulatory, and ultimately increase survival of young by increasing the chance of fertilization by a high‐quality or genetically compatible male (Watson, 1991; Yasui & Garcia‐Gonzalez, 2016; Zeh & Zeh, 2001). The simplest hypothesis proposed, and the one we test in the current study, is the encounter rate hypothesis (ERH), which states that the rate of multiple mating increases with an increase in frequency of encounters between mature males and receptive females during the mating season (Boomer et al., 2013; Daly‐Engel et al., 2006, 2007, 2010; Nosal et al., 2013). According to the ERH, the more time available for mating between broods, the higher the predicted rate of multiple paternity.

**FIGURE 1:**
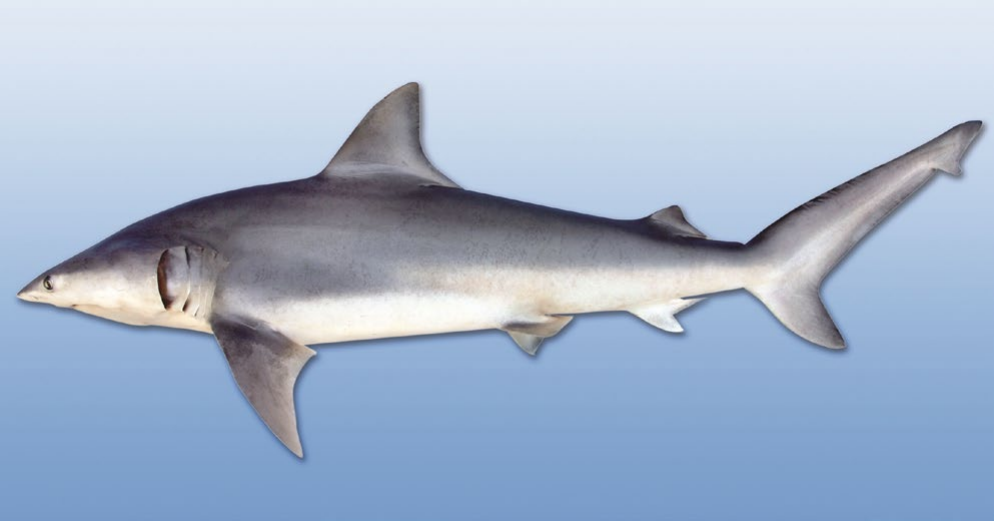
The finetooth shark, *Carcharhinus isodon*

We tested the ERH by estimating multiple paternity rates in two populations of the finetooth shark (*Carcharhinus isodon*; Figure 1), a small coastal requiem shark that uses placental viviparity to reproduce (Castro, 1993; Compagno et al., 2005). The most recent U.S. stock assessment, conducted in 2007, characterizes the finetooth shark in the northwestern Atlantic Ocean and Gulf of Mexico (GoM) as a single stock (NOAA, 2007), though genetic analysis has shown significant population structure between Atlantic and GoM populations, indicating little historical migration between ocean basins (Portnoy et al., 2016). Population‐level differences abound between these regions; as a result of warmer waters in the GoM, finetooth sharks in the GoM do not migrate as far as conspecifics on the Atlantic coast (Castro, 1993; Driggers & Hoffmayer, 2009; Drymon et al., 2010), while finetooth sharks mature more slowly in the northwestern Atlantic than they do in the GoM (Higgs et al., 2020; Vinyard et al., 2019), potentially facilitating differences in fecundity, growth rate, and mating strategy (Carlson et al., 2003).

As with many members of this speciose genus, parturition in the finetooth shark occurs in early spring after a gestation period of approximately one year and is thought to be followed by a “resting year” in which the animal does not reproduce (Brown et al., 2020; Castro, 1993; Higgs et al., 2020). In a study by Castro (1993), a cohort of reproductively mature female finetooth sharks caught on the Atlantic coasts of South Carolina and Florida over a period of 10 years was found to consist of ~50% gravid females carrying full‐term pups and ~50% nongravid females carrying ripe oocytes, a split indicative of a biennial reproductive cycle. This was recently confirmed by Brown et al. (2020) on a sample of 88 mature females collected in 2012–2015 in the same region. However, a study by Driggers and Hoffmayer (2009) reported seven mature finetooth shark females in the GoM, two of which displayed characteristics of annual reproduction as indicated by the presence of both near‐term embryos and mature oocytes in the same individual. The authors postulated that some sharks may switch from biennial to annual reproduction as a result of energy allocation; full‐grown individuals with adequate food supply that are not required to migrate long distances could allocate that energy to increasing their frequency of reproduction. Since then, a study with a larger sample of females (*n* = 50) captured during the peak ovulation/parturition period of (April–June) confirmed the presence of both reproductive periodicities in the northern GoM, showing approximately a 65:35 ratio (32 annual, 18 biennial) among females (Higgs et al., 2020). Because such divergence in reproductive periodicities is unusual among sharks (Driggers & Hoffmayer, 2009), this species represents a rare opportunity to examine the ecological dependence and local adaptive value of multiple paternity, including potential effects on population genetic diversity.

In this study, we compared rates of multiple paternity and standing gene diversity (including allelic richness) between two populations of sharks with varying reproductive periodicities using 12 highly polymorphic microsatellite DNA loci. Little is directly known about sperm storage and competition in most sharks, including the finetooth shark, but females from many shark species are capable of storing sperm in the oviducal gland for months to years before eggs are fertilized (Pratt, 1993), and sperm competition in response to polyandry has been shown to drive selection on reproductive traits in sharks and bony fishes (Rowley et al., 2019; Rowley, Locatello, et al., 2019). Given the ubiquity of multiple paternity in elasmobranchs in general and the genus *Carcharhinus* in particular (Byrne & Avise, 2012; Fitzpatrick et al., 2012), plus the fact that female finetooth sharks can likely store sperm from multiple conspecifics and in the absence of data on sperm competition, we assume here that any male they mate with could genetically contribute to the next litter. Finetooth sharks aggregate and mate in May–June and have a gestation period of 11–12 months (Castro, 1993; Higgs et al., 2020), so we assume that under the ERH, a female shark with biennial reproduction could participate in twice as many mating periods between litters compared with an annual reproducer, resulting in a correspondingly higher rate of multiple paternity. We therefore hypothesize that the overall frequency of MP would be lower for finetooth shark females in the GoM, where some individuals are annual reproducers, and higher in the Atlantic, where all individuals are biennial reproducers. We further predict that MP will be lower among annual reproducers in the GoM compared with biennial conspecifics in the same population, which could result in a loss of diversity in the region with lower MP.

## 2 MATERIALS AND METHODS

Gravid female finetooth sharks were collected from the northern GoM between Apalachicola Bay, Florida and East Bay, Louisiana, by longline and gillnet in 2011–2013, and from the northwest Atlantic on the coast of South Carolina (hereafter Atlantic) by longline and gillnet in 2014–2016. Reproductive periodicity was determined following the methods described in Higgs et al. (2020) in females caught during the peak mating/parturition period, with females exhibiting simultaneous vitellogenesis and gestation being classified as reproducing annually. Small (~1 cm^3^) fin or muscle samples were obtained from the gravid females and each in utero pup and were stored in 1.5 ml DMSO buffer or >75% ethanol. DNA extraction was done via a salting‐out procedure adapted from Sunnucks and Hales (1996). The genetic mating system of the finetooth shark was assessed using adult female DNA from 98 GoM and 30 Atlantic specimens. A suite of microsatellite markers specific to finetooth sharks (Giresi et al., 2012a) plus loci cross‐amplified from congeners *C. acronotus* (blacknose shark; Giresi et al., 2012b) and *C. amblyrhynchos* (gray reef shark; Momigliano et al., 2014) were employed. The M13‐tailed primer protocol from Boutin‐Ganache et al. (2001) was adapted using primers obtained from Integrated DNA Technologies (Coralville, IA). PCR methods were as follows: (a) initial denaturation at 95℃ for 4 min; (b) 30 cycles consisting of 1 min. at 95℃, 30 s. at optimal annealing temperature, and another 30 s. at 72℃; and (c) 20 min of extension at 72°C. The resulting PCR product was then visualized via electrophoresis and sent to the University of Arizona Genetics Core (UAGC, Tucson, AZ) for fragment analysis in an ABI3730 DNA Analyzer. Fragment analysis results were visually scored using the microsatellite plug‐in for the program GENEIOUS v. 9.1.5 (Kearse et al., 2012).

The Markov chain method in the program GENEPOP (Raymond & Rousset, 1995) was used to estimate observed and expected heterozygosity, to test for deviation from Hardy–Weinberg equilibrium, and to calculate the allele frequencies at each locus using DNA from the adult sharks. The program MICRO‐CHECKER v. 2.2.3 (Van Oosterhout et al., 2004) was used to detect genotyping errors due to null alleles, large allele dropout, stuttering, and pipetting error, and molecular indices of diversity were calculated in Arlequin v. 3.5 (Excoffier & Lischer, 2010).

Alleles in mothers and offspring were scored visually using GENEIOUS, and any litters with more than two nonmaternal alleles at two or more loci indicated the presence of multiple sires. Litters showing multiple paternity at only one locus were not included. This method of scoring serves as a conservative baseline because it assumes that every male in the population is a heterozygote at every locus, which is unlikely in elasmobranchs, which have naturally low rates of molecular evolution compared with other taxa (Martin et al., 1992). Additional methods for paternity analysis included the programs FMM (Frequency of Multiple Mating; Neff et al., 2002), which uses Bayesian priors that account for population allele frequencies to generate a 95% confidence interval for frequency of MP, and PrDM (Probability of Detecting Multiple mating; Neff & Pitcher, 2002), which calculated the power of our locus set to detect MP in litters of varying sizes and in the presence of paternal skew. We tested three paternal contribution scenarios: (a) two sires with even skew (0.5:0.5), (b) two sires with moderate skew (0.33:0.67), and (c) two sires with high skew (0.1:0.9). This is particularly important in this study because of the small mean litter size; the probability of detecting multiple mating is greatly increased when litter size increases, and it is likely that sperm competition plays a role in the mating strategy of the finetooth shark, similar to other sharks (Portnoy et al., 2007; Rowley, Daly‐Engel, et al., 2019; Rowley, Locatello, et al., 2019). To avoid potential type II error, a “scaled MP” value was calculated for each population grouping based on mean litter size and paternal skew, in order to estimate the rate of MP if our probability of detection was 100%. Specifically, the scaled value for each population was calculated as the MP rate according to FMM divided by the PrDM value for the average litter size for that population, to reflect how much more multiple mating might be detected if PrDM = 1.

The program GERUD v.2.0 (Jones, 2005) was used to estimate the number of sires in a brood across all loci simultaneously by reconstructing parental genotypes from the genotypes of the progeny. The program COLONY v.2.0.6.4 (Jones & Wang, 2010) was used to extract parentage and sibship information from genotype data using a full likelihood method. As these programs reconstruct paternal genotypes from the offspring data and assign parentage, they can also show whether reproductive skew has occurred using genotype reconstruction or maximum likelihood, respectively.

Molecular indices of diversity among unrelated individuals were generated in FSTAT v1.2 (Goudet, 1995). These included Nei’s (1987) unbiased estimator of gene diversity (*H*) and allelic richness (*A*_r_), which provides a measure of allelism that is corrected for and independent of sample size, allowing for accurate comparison among groupings with unequal sampling. Paired *t* tests were used to determine whether differences in diversity metrics across loci were significant between the Atlantic and GoM, and between annual and biennial reproducers. Fisher's exact test was performed using SAS Software, version 9.4 (SAS Institute, Inc., Cary, NC) to detect significant variation between groups. Statistical significance was defined a priori as *p* < .05.

## 3 RESULTS

The mean litter size was 4.02 (*SD* = 1.04) pups in the GoM and 4.06 (*SD* = 0.64) in the Atlantic, and brood size ranged between 1 and 9 individuals. To avoid extrapolation and ensure our ability to detect multiple paternity, only gravid females with three or more pups were analyzed (Daly‐Engel et al., 2007). This included 92 of the 98 adults and their pups from the northern GoM (*N* = 481 individuals) and 17 of the 30 adults and their pups from the northwest Atlantic (*N* = 86 individuals). Reproductive periodicity was known for 34 of the females from the GoM (biennial *N* = 10, annual *N* = 24); all females in the Atlantic were reproducing biennially (*N* = 30).

No significant deviation from Hardy–Weinberg equilibrium and no evidence of genotyping errors, including null alleles, large allele dropout, or pipetting error, were found at the 12 loci used in this study (Table 1). With a mean litter size of approximately four pups, the PrDM output for four offspring provides the nearest estimation of the actual detection probability for the population overall. The average probability of detecting multiple paternity was estimated to be between 33% (for high skew) and 85% (for even skew), depending on the ratio of genetic contribution. COLONY and GERUD results indicated no evidence of paternal skew in any litter (e.g., litters of four pups with two sires primarily had a ratio of 2:2, and the largest litter of nine pups had three sires with a ratio of 2:3:4 pups per sire), so even (0.5:0.5) male contribution was assumed, for which the PrDM program gave a probability of detecting multiple mating of 85%.

**TABLE 1:** Descriptive statistics for microsatellite loci used in this study

Locus	Size range	*k*	*H*_o_	*H*_e_	HWE *p*‐values	Source
Cac67	177–251	31	0.9655	0.9591	.7051	Giresi et al. (2012b)
Cam15	224–240	8	0.5000	0.5008	.5689	Momigliano et al. (2014)
Cis102	224–236	4	0.6146	0.5196	.1144	Giresi et al. (2012a)
Cis107	292–310	10	0.8404	0.8091	.1989	Giresi et al. (2012a)
Cis108	272–280	5	0.4444	0.4833	.6258	Giresi et al. (2012a)
Cis111	166–170	3	0.4316	0.4556	.5959	Giresi et al. (2012a)
Cis121	241–265	11	0.5684	0.5496	.2640	Giresi et al. (2012a)
Cis131	308–316	4	0.6129	0.6140	.5093	Giresi et al. (2012a)
Cis157	230–246	8	0.5584	0.5039	.5716	Giresi et al. (2012a)
Cis161	192–222	15	0.9535	0.8897	.6407	Giresi et al. (2012a)
Cis168	158–162	3	0.3878	0.3846	1.0000	Giresi et al. (2012a)
Cis175	210–246	17	0.8913	0.8614	.5413	Giresi et al. (2012a)

Note

Size range is given in base pairs. *k* = number of alleles; *H*_o_ and *H*_e_ denote observed and expected heterozygosity, respectively.

The most conservative estimate of MP based on visual scoring showed 53 out of 92 litters from the GoM having three or more paternal alleles at two or more loci, giving an overall estimated minimum frequency of MP of 57.6%. An additional 11 litters had three or more paternal alleles at only one locus, which was not considered sufficient to demonstrate MP under this method. The remaining 28 litters had no evidence of MP. In the Atlantic population, 10 out of 17 litters had three or more paternal alleles at two or more loci, giving a minimum expected frequency of MP of 58.8%. A further five litters had three or more paternal alleles at only one locus, and two litters had no evidence of multiple paternity. Within the GoM, frequency of MP based on visual scoring of annual litters was estimated to be 67% (16 of 24 litters), while frequency of MP among biennial litters was 70% (7 of 10 litters).

The program FMM, which takes into account the prior distribution of alleles in the population, estimated the frequency of multiple mating at 83.0% in the GoM population (95% CI: 77%–97%), and 88.2% in the Atlantic (95% CI: 61%–98%). The frequency of multiple mating among females reproducing on a biennial cycle in the GoM was 76.6% (95% CI: 41%–98%) and among females on an annual cycle was 93.0% (95% CI: 77%–98%). As these were considered the most reliable results, the calculations of scaled MP were based on this method (Table 2). GERUD results indicated that the largest litter of nine pups was sired by a minimum of three males, while only two sires were detected in all other multiple‐sired litters. COLONY full likelihood results, considered the least conservative, estimated 100% multiple mating in both populations. There was no significant correlation between litter size and rate of MP (*p* > .05).

**TABLE 2:** Summary mating system statistics

Population	Number of litters	Mean litter size	MP (%)	95% CI	Scaled MP (%)	*H*	*A*_r_
GoM	92	3.91	83.0	77–97	97.6	0.628 ± 0.057	4.916 ± 0.906
Atlantic	17	4.06	88.2	61–98	100	0.633 ± 0.061	4.680 ± 0.794
GoM annual	24	3.95	93.0	77–98	100	0.624 ± 0.064	5.06 ± 1.01
GoM biennial	10	3.81	76.6	41–98	90.1	0.617 ± 0.058	4.66 ± 0.925

Note

MP, Percent of multiple paternity calculated in FMM; 95% CI, confidence intervals for FMM; Scaled MP, rate of MP if detection probability was 100%; *H*, gene diversity; *A*_r_, allelic richness; GoM, Gulf of Mexico.

Fisher's exact tests showed no significant difference in rates of MP between the two periodicities within the GoM, or between the samples from the GoM population and the Atlantic population, including scaled and unscaled values of MP (*p* > .05). Similarly, no evidence was found that estimates of gene diversity (*H*) or allelic richness (*A*_r_) across 12 microsatellite loci varied significantly between any two groups in our study. Between the northwest Atlantic (*H* = 0.633 ± 0.061, *A*_r_ = 4.680 ± 0.794) and GoM (*H* = 0.628 ± 0.057, *A*_r_ = 4.916 ± 0.906), there was no significant variation (*P*_H_ = 0.952; *P*_Ar_ = 0.846), nor between annually (*H* = 0.624 ± 0.064, *A*_r_ = 5.06 ± 1.01) and biennially (*H* = 0.617 ± 0.058, *A*_r_ = 4.66 ± 0.925) reproducing finetooth sharks within the GoM (*P*_H_ = 0.943; *P*_Ar_ = 0.772; Table 2).

## 4 DISCUSSION

Using Bayesian prior analysis, we detected an 83.0% frequency of multiple mating in 92 litters of the finetooth shark in the GoM and an 88.2% frequency in 17 litters in the northwest Atlantic. Females in the GoM reproducing on an annual cycle had an MP frequency of 93.0%, and those on a biennial cycle had an MP frequency of 76.6%. These frequencies were statistically similar across geographic regions, but not significantly different between varying periodicities within the GoM. Similarly, no significant differences were identified in diversity metrices by geographic or reproductive population (Table 2), despite the presence of historical barriers to gene flow dividing the northwest Atlantic from the GoM (Portnoy et al., 2016). Despite high polyandry, we found no evidence for increased fitness as a result of multiple mating; also, as in previous studies on sharks (Boomer et al., 2013; Daly‐Engel et al., 2010; Portnoy et al., 2007), litter size and rate of MP were not correlated.

Though a predominance of polyandry was detected among female finetooth sharks, the frequency of MP varied somewhat between the different methods used in this study. Visual scoring is the most conservative method because it assumes that males are heterozygous at all loci, which may undercount MP at loci with low polymorphism, and may have accounted for the difference observed in this study. However, FMM takes into account the number of microsatellite loci, their degree of polymorphism, and the number of pups in each litter to provide a less conservative estimate of multiple mating. While these numbers differ, it is apparent that a majority of females in this population are polyandrous, with an above‐average frequency compared with other shark species studied to date (for a review, see Rossouw et al., 2016). Furthermore, given the low probability of detecting multiple mating in smaller litters, our unscaled results are more likely an underestimation than an overestimation.

There are no documented observations of mating in finetooth sharks, but studies have shown that this species aggregates in large numbers (Castro, 1993). Castro (1993) reported seasonal migration of this species between the waters of South Carolina and Florida along the East Coast, where large numbers of both adults and juveniles were caught. Though no seasonal migrations of finetooth sharks within the GoM have been explicitly identified, research has shown the occurrence of seasonal concentrations of adult finetooth in coastal waters (Parsons et al., 2007; Bethea et al., 2014). The ERH (Daly‐Engel et al., 2007) seeks to explain rates of MP based on how often receptive females encounter and mate with mature males in the absence of any detectable benefit to females and depending on how frequently females are receptive versus resting. Under the ERH, aggregative behavior may increase the number of mating opportunities and/or result in mobbing or crowding behavior, which can also increase MP (Daly‐Engel et al., 2007). Mobbing or crowding, which occurs when multiple males simultaneously attempt to coerce mating with a single female, has been observed in nurse sharks (Pratt & Carrier, 2001) and whitetip reef sharks (Whitney et al., 2004). By comparison, recent studies on multiple paternity in the tiger shark—a large, pelagic species in which aggregations have not been observed—showed an absence of MP, both in four litters from the east coast of Australia (Holmes et al., 2018) and four from the western Indian Ocean (Pirog et al., 2020). Support for increased multiple mating among organisms that aggregate more densely can also be found among other taxa, such as sea turtles (for a review, see Lee et al., 2018). If finetooth sharks mate in large aggregations, it could drive high rates of convenience polyandry, where a female concedes to mating to avoid harm (DiBattista et al., 2008), especially if only a fraction of the females of the population are mating in a given year. In contrast, if female choice is operating and biennially reproducing females choose not to mate during their resting year, then the reproductive periods are roughly equivalent between annual and biennial populations and rates of MP are unlikely to vary.

With no significant difference detected between reproductive periodicity, frequency of multiple paternity, or genetic diversity in these two populations, we were unable to find support for the ERH, but the notion warrants further scrutiny. Reproductive periodicity may be a poor indicator of the rate of multiple paternity, which may imply that females in both categories are facing the same general mating conditions (e.g., aggregation density, timing of mating). It is difficult to estimate how behavioral differences in female receptivity might vary between populations, but genetic data show no sex‐biased movement in this species, suggesting that males and females in both regions are broadly philopatric (Portnoy et al., 2016). With current methods, it is not possible to follow individual females from year to year to determine whether they reproduce annually, biennially, or switch between the two based on resource availability. Little is known about sperm competition in female finetooth sharks, but evidence of sperm storage has been found in several related species, including the dusky shark (*Carcharhinus obscurus*; Pratt, 1993), smooth‐hound (*Mustelus canis*; Hamlett et al., 2002), and gummy shark (*Mustelus antarcticus*; Storrie et al., 2008). Sperm competition may lead to difficulties detecting polyandry with genetic techniques, particularly if insemination is heavily skewed toward one male (Neff & Pitcher, 2002). The GoM population of finetooth sharks showed no evidence for reproductive skew, which could indicate a lack of long‐term sperm storage and/or postcopulatory sexual selection. However, it is possible that females mating on an annual cycle have little to no paternal skew due to a lack of time for sperm competition to occur. Females on a biennial cycle that do not take a resting year, on the other hand, could mate immediately following parturition and store sperm throughout their postpartum year, allowing more time for postcopulatory sexual selection (Fitzpatrick et al., 2012; Rowley, Daly‐Engel, et al., 2019; Rowley, Locatello, et al., 2019).

## 5 CONCLUSION

As marine apex and mesopredators, sharks are one of the most important taxonomic groups in marine ecosystems, helping to maintain the diversity of ocean habitats from shallow coastal waters to the deep sea. Because multiple mating appears to be common in elasmobranchs, there may be some ultimate genetic benefit that allows for increased adaptability in these long‐lived vertebrates. In general, understanding mating systems is crucial for estimating population viability in elasmobranchs with varying life‐history characters, as many populations are faced with local decline. For finetooth sharks in particular, we recommend further investigation into the mechanisms that cause variation in reproductive periodicity, which may affect other aspects of shark mating systems. The current federal fishery regulations in the United States determine commercial and recreational take limits for all small coastal shark species, including finetooth sharks in both the GoM and coastal Atlantic (NOAA, 2007). However, marked differences in reproductive periodicity and genetic diversity, among other traits, indicate that each population may require separate management.

## CONFLICT OF INTEREST

None declared.

## AUTHOR CONTRIBUTIONS

**Cody S. Nash:** Data curation (equal); formal analysis (lead); funding acquisition (supporting); investigation (lead); visualization (equal); writing–original draft (lead); writing–review and editing (equal). **Philip C. Darby:** Funding acquisition (equal); resources (supporting); writing–review and editing (equal). **Bryan S. Frazier:** Investigation (supporting); resources (equal); writing–review and editing (equal). **Jill M. Hendon:** Investigation (supporting); resources (equal); writing–review and editing (equal). **Jeremy M. Higgs:** Investigation (supporting); resources (equal); writing–review and editing (equal). **Eric R. Hoffmayer:** Investigation (supporting); resources (equal); writing–review and editing (equal). **Toby S. Daly‐Engel:** Conceptualization (lead); data curation (supporting); formal analysis (supporting); funding acquisition (equal); methodology (equal); project administration (equal); resources (equal); supervision (equal); writing–original draft (supporting); writing–review and editing (equal).

## Data Availability

All primers and analyses used in this study have previously been published and are publicly available.
